# Systems approach to monitoring and evaluation guides scale up of the Standard Days Method of family planning in Rwanda

**DOI:** 10.9745/GHSP-D-13-00165

**Published:** 2014-05-04

**Authors:** Susan Igras, Irit Sinai, Marie Mukabatsinda, Fidele Ngabo, Victoria Jennings, Rebecka Lundgren

**Affiliations:** a Georgetown University's, Institute for Reproductive Health, Washington, DC, USA; b Institute for Reproductive Health, Kigali, Rwanda; c Ministry of Health [Rwanda], Kigali, Rwanda

## Abstract

There is no guarantee that a successful pilot program introducing a reproductive health innovation can also be expanded successfully to the national or regional level, because the scaling-up process is complex and multilayered. This article describes how a successful pilot program to integrate the Standard Days Method (SDM) of family planning into existing Ministry of Health services was scaled up nationally in Rwanda. Much of the success of the scale-up effort was due to systematic use of monitoring and evaluation (M&E) data from several sources to make midcourse corrections. Four lessons learned illustrate this crucially important approach. First, ongoing M&E data showed that provider training protocols and client materials that worked in the pilot phase did not work at scale; therefore, we simplified these materials to support integration into the national program. Second, triangulation of ongoing monitoring data with national health facility and population-based surveys revealed serious problems in supply chain mechanisms that affected SDM (and the accompanying CycleBeads client tool) availability and use; new procedures for ordering supplies and monitoring stockouts were instituted at the facility level. Third, supervision reports and special studies revealed that providers were imposing unnecessary medical barriers to SDM use; refresher training and revised supervision protocols improved provider practices. Finally, informal environmental scans, stakeholder interviews, and key events timelines identified shifting political and health policy environments that influenced scale-up outcomes; ongoing advocacy efforts are addressing these issues. The SDM scale-up experience in Rwanda confirms the importance of monitoring and evaluating programmatic efforts continuously, using a variety of data sources, to improve program outcomes.

## Abstract (precis)

Scaling-up lessons included: (1) simplifying provider training and client materials; (2) ensuring core aspects of the intervention, for example, that the CycleBeads client tool was integrated into the supply chain system; (3) addressing provider-generated medical barriers; and (4) managing threats from changing political and policy environments. A focus on systems, the use of multiple M&E data sources, maintaining fidelity of the innovation, and ongoing environmental scans facilitated scale-up success.

## BACKGROUND

Rwanda is the most densely populated country in Africa and one of the poorest countries in the world.^1^ Following the devastating 1994 genocide, the country made intensive efforts to improve its social, economic, and health conditions. But almost 2 decades later, the health system still faces many challenges, including meeting people's reproductive health needs. In 2005, the total fertility rate was more than 6 children per woman, and almost 40% of women of reproductive age had an unmet need for modern contraceptive methods.^2^

In an effort to help women meet their contraceptive needs and achieve healthy timing and spacing of pregnancies, the Rwanda Ministry of Health (MOH) joined in partnership with the Institute for Reproductive Health (IRH) at Georgetown University, as well as with other pri<@?show=[fo]?>vate and faith-based health groups, to expand access to the Standard Days Method (SDM) throughout the country.

SDM is a fertility awareness-based method of family planning based on a woman's menstrual cycle (Box). Because SDM is a low-cost fertility awareness-based method with no side effects, was acceptable to faith-based groups, and does not require follow-up visits or resupplies, it filled a special niche in the Rwandan family planning program.

The Standard Days Method is an inexpensive, fertility awareness-based method with no side effects, and it does not require follow-up visits or resupplies.

**Box. What is the Standard Days Method?**

The Standard Days Method (SDM) is a simple, fertility awareness-based method of family planning developed and tested by Georgetown University's Institute for Reproductive Health. Based on reproductive physiology, SDM identifies the days in the menstrual cycle (days 8–19) when a woman can get pregnant if she has unprotected sex. CycleBeads, a color-coded string of beads, helps women track the days of their cycles when they are most likely to get pregnant. The method works best for women with cycles that usually range 26–32 days. Over half of women meet this criterion.

If the woman does not want to get pregnant, she and her partner avoid unprotected sex on days 8 through 19 of her cycle. An efficacy study found a failure rate for SDM of 5 per 100 woman-years when used correctly. The failure rate during typical use is 12 per 100 woman-years.^3^

SDM has been introduced and assessed in different facility and community-based service delivery settings for over 12 years.^4^ The U.S. Agency for International Development and the World Health Organization have globally recognized the method as a modern, evidence-based contraceptive practice,^5^ and it is currently offered in more than 30 countries.

The method had been integrated successfully into both clinical and community-based government services in pilot programs. Scaling up the pilot program to the entire country, however, was a complex task. International family planning research shows that unless a new method is introduced in a systematic and strategic way, results are not likely to be positive or sustainable.^6^^,^^7^ For scale up to be successful, understanding the changing environmental contexts in expanded geographic areas—which may differ in significant ways from the pilot sites—is critical,^8^ and the concerns of many key stakeholders must be addressed.^9^ Partner organizations are essential to expand access and to leverage technical and financial resources, but they often have different project and funding durations from the scale-up program.

Developing workforce capacity to offer the new family planning method as part of routine service delivery is at the heart of scaling up, but it takes multiple family planning actors to make this happen, each with varying roles, abilities, and resources to apply to the scale-up process. New methods must be included in supply chain systems, and it can take several years before changes become operationalized in periphery services. Budget allocations for a new method require advocacy and evidence to reassure policy makers during scale up that the program investment is worthwhile. New methods are not yet well-integrated into routine monitoring and evaluation (M&E) systems in the early stage of scale up, so additional information sources are required to monitor the pace of expansion and integrity of the innovation. Thus, the process of wide-scale integration of the new method within a complex health system cannot be controlled or monitored to the same extent as more localized introduction efforts during the pilot stage.

It takes time to integrate new contraceptive methods into routine M&E systems, so additional data sources are often needed to monitor scale up.

To inform our scale-up process, we adopted the principles of the World Health Organization (WHO)/ExpandNet conceptual framework for sustainable scale up,^10^ the corollary Nine-Step Guide to develop a strategic scale-up plan,^11^ and related guidance by Simmons and Shiffman,^12^ who summarize the characteristics of a good scaling-up strategy, based on diffusion of innovation theory^13^ and other literature on scaling up health practices. Such characteristics comprise:

- An intervention that can be adapted to fit into the existing health system
- A participatory approach that includes local and central stakeholders and policy makers
- Reliance on systematic use of evidence for decision-making
- An ongoing focus on sustainability

After providing a brief introduction about the outcomes of the pilot phase as well as goals and outcomes of national scale up, this article provides lessons learned about how to successfully scale up health interventions. These lessons demonstrate the importance of ongoing monitoring and evaluation efforts for making midcourse corrections that support successful scale up.

## SDM INTRODUCTION AND SCALE UP IN RWANDA

### Pilot Phase Demonstrates Demand for SDM

In 2002, we introduced SDM in Rwanda through a pilot program in 7 public health facilities, 5 clinics run by faith-based organizations, and 1 nongovernmental organization site. In 2004, we introduced SDM in 15 more facilities (Figure).

**FIGURE.**
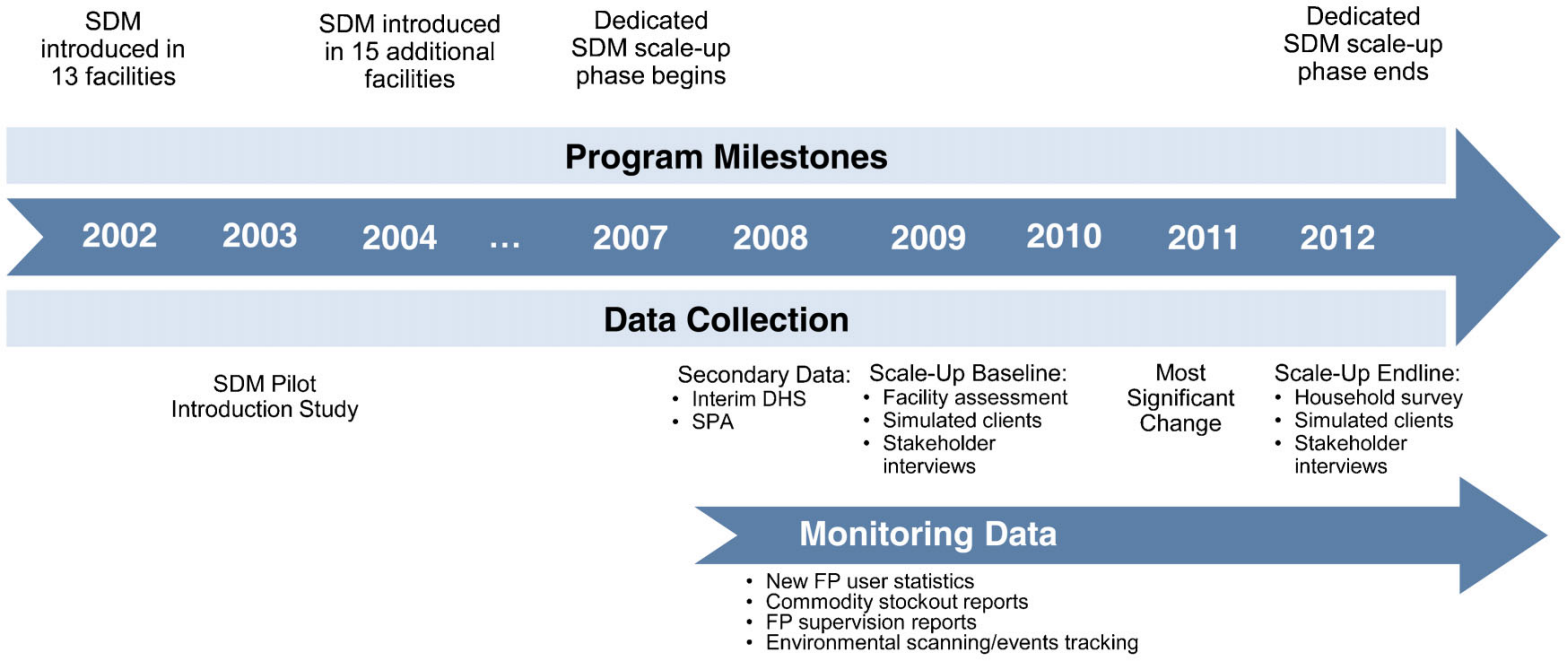
SDM Program Milestones and Data Collection Timeline, Rwanda, 2002–2012 Abbreviations: DHS, Demographic and Health Survey; FP, family planning; SDM, Standard Days Method; SPA, Service Provision Assessment.

The pilot program generated substantial demand for SDM: service statistics showed that 23% of new method users chose SDM.^14^ Interviews and focus groups confirmed that the method was easy to offer by providers, was a viable choice for many couples, and was often adopted by women who had never before used a modern method. Offering SDM also had an additive effect on contraceptive prevalence rates,^14^ making it an attractive option for the Rwandan national family planning program.

During the pilot phase, 23% of new contraceptive method users chose the Standard Days Method.

### Scale-Up Challenges and Goals

Between 2005 and 2007, the country revitalized family planning efforts, and the MOH took this opportunity to integrate SDM into the new family planning policies, norms, training curricula, and management information and logistics systems.

Within this favorable policy environment, geographic expansion of SDM services continued in 2007 under a 6-year, dedicated scale-up program. Considerable progress had been made already in both horizontal scale up (geographic expansion) and vertical scale up (institutionalization, such as, inclusion in norms, training, supervision, procurement, and reporting systems). But much work remained:

- The program had to **expand to the many districts** where SDM was not yet available and **build the capacity** of national and local organizations to offer the method without outside technical assistance.
- SDM had to be integrated into **preservice training**—a key element of sustainability.
- The revised family planning policies had to be operationalized so that CycleBeads, a tool to help women track their fertile and infertile days, and related instructional materials would be included in **supply chains**, and so that SDM would become part of **routine service statistics**.
- Even though there was top-level approval, scale up required **advocacy** to create support among policy makers and service providers at different levels for adding a new family planning method.
- Scale up also relied on **mass media and community-level promotion** to ensure potential clients knew of the new method option, its unique attributes compared with other methods, and where to find facilities that offered it.

The Rwanda MOH continued its close involvement with SDM scale up throughout the country via the Maternal and Child Health Task Force and its subsidiary Family Planning Technical Committee, made up of key family planning actors including MOH, donors, and international and national nongovernmental and faith-based organizations. End-of-project goals identified by partners and key stakeholders included:

1. Availability of SDM in 95% of public and private health facilities that offer family planning and in all community-based family planning services
2. Institutionalization of SDM into family planning support systems

To manage the complex set of actions required, the partners developed a strategic plan to achieve these goals over 6 years, which encompassed strategic planning and coordination of organizational roles, phased-in implementation of activities, M&E, and midcourse corrections throughout the process.[Fig f02]

**Figure f02:**
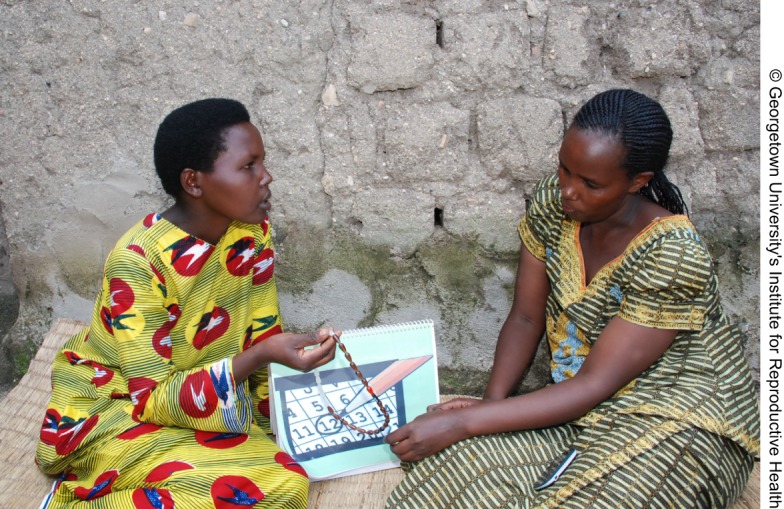
A health care provider shows a client how to use the Standard Days Method of family planning with CycleBeads.

### Scale-Up Outcomes

The dedicated scale-up effort using a systems lens led to near-nationwide availability of SDM by the end of the scale-up period. In fact, by the end of the scale-up project, 717 service delivery points included SDM in the method mix, surpassing the benchmark of 690, and more than 7,000 individuals had been trained to counsel clients on how to use SDM (Table 1). According to endline survey results, awareness of the method among women and men was on the same level as other, more established methods, and 7.4% of women using family planning chose to use SDM. Most women using SDM at the time of the survey were satisfied with the method (97.5%) and planned to continue using it (87.4%).

**TABLE 1.** First-Year and End-of-Project Outcomes Compared With Benchmarks

**Indicator**	**First Year (2007)^a^**		**End of Project (2012)**		**5-Year Benchmarks**
	**No.**	**% of Benchmark**	**No.**	**% of Benchmark**	
Service delivery points that include SDM in the method mix	356	51.2	717	103.9	690
Individuals trained to counsel clients on how to use SDM	1,679	31.0	7,472	138.4	5,400
Organizations that have capacity to undertake SDM activities	5	50.0	7	70.0	10
Essential or key policies, norms, guidelines, and protocols in which SDM is included	2	50.0	3.5	87.5	4
Public or private training organizations that include SDM in their preservice training and/or continuing education	5	100.0	5	100.0	5
Public or private training organizations that include SDM in their in-service training	4	40.0	7	70.0	10
Information, education, and communication activities, materials, and mass media that include SDM	7	58.3	12	100.0	12

Abbreviation: SDM, Standard Days Method.

a Includes SDM pilot activity in the country starting in 2002.

## LESSONS LEARNED FROM MONITORING AND EVALUATING THE SCALE-UP PROCESS

Because scale up is a non-linear process that occurs within complex systems with engagement of multiple organizations and health system actors, strategic use of data from multiple sources throughout the scale-up process provides timely information to allow program corrections and to support the policy process. To provide useful information, our M&E efforts had to cut across multiple levels, sources, and phases (Table 2). Lessons learned about our scale-up process follow, demonstrating the importance of collecting and using data to make midcourse corrections that supported successful scale up.

**TABLE 2.** Monitoring and Evaluation Data Collection by Scale-Up Indicator

**Indicator**	**M&E Method^a^**	**Type of Data**	**Main Purpose**	**Timing**
**Outcomes**	Household survey	Quantitative	Evaluation	Endline
• Awareness and use of SDM	Service statistics	Quantitative	Monitoring	Monthly
• Availability of quality services	“Most Significant Change” story collection	Qualitative	Evaluation	Year 4
• Provider competency	Provider supervision and client follow-up reporting	Quantitative	Monitoring	Ongoing
	Simulated clients study	Quantitative	Evaluation	Baseline and endline
**Outputs**	Facility/service delivery point survey	Mixed	Evaluation	Baseline
• Providers trained	Stakeholder interviews	Qualitative	Evaluation	Baseline and endline
• Clinics offering SDM	Benchmark reporting	Quantitative	Monitoring	Semiannually
• Demand-oriented Information, Education and Communication (IEC)				
• Supportive partners/stakeholders				
• Systems integration				
**Process**	Staff assessments of data on scale-up status	Qualitative	Monitoring	Annually
• Scale-up strategy	Organizational capacity assessments	Qualitative	Evaluation	Ongoing
• Dissemination and advocacy	Environmental scanning, including key events timeline reporting	Qualitative	Monitoring	Ongoing
• Organizational capacity-building process				
• Resource mobilization				
• Environmental influences				

Abbreviations: M&E, monitoring and evaluation; SDM, Standard Days Method.

a Classification based on method's main M&E contribution, although there is overlap; for example, stakeholder interviews also assessed environmental influences, and resource mobilization was documented as part of benchmarking.

### Lesson Learned 1. Expect to simplify elements of the intervention—even if they worked in the pilot phase—to function at scale and to ensure sustained integration into existing systems.

Results of provider supervision and client follow-up visits revealed that providers and clients at the scale-up sites found the training protocols and client materials from the pilot phase too difficult to use. We realized that the SDM intervention needed to be simplified further to support its integration into the national family planning program, since we could not provide the same concentrated attention to the larger number of facilities and community settings as we did to the smaller number of pilot sites.

Training protocols and counseling materials had to be simplified for national scale up.

We then field-tested the resulting simplified user instructions, translated into Kinyarwanda (the native language in Rwanda), to ensure that providers counseled accurately and that clients received correct information using the modified instructions. Client materials were modified a second time in preparation for including SDM in social marketing within private-sector pharmacies and clinics.

### Lesson Learned 2. Maintain integrity of core aspects of the innovation package.

M&E efforts also exposed the importance of defining the intervention “package” clearly—in terms of ensuring both successful scale up and accurate assessments of availability of the package. Although some components of pilot projects must be adapted as mentioned under the first lesson learned, critical aspects of the intervention must remain intact for scale up. According to the partners' definition, the core SDM package included CycleBeads (offered in a small plastic bag with instructions and a multi-year calendar), training curricula and in-service training materials for health care providers and supervisors, and awareness-raising materials and activities that focused on both men and women.

Assessment of data from multiple sources, including ongoing program monitoring data as well as national health facility and population-based surveys, revealed seemingly incompatible data findings about SDM availability in facilities and use among women. As it turned out, the national surveys used a different definition of the full SDM package, which made a substantial difference in SDM availability and use.

Specifically, according to the preliminary Rwandan Service Provision Assessment (SPA) issued in 2008,^15^ 75% of facilities that offered family planning reported offering SDM—25% more than our scale-up monitoring data had indicated. However, interim Demographic and Health Survey (DHS)^16^ data found that while 64% of women had heard of SDM, only 0.3% of women said they were using it (Table 3). So although most facilities were seemingly offering SDM and most women had heard of the method, very few women were actually using it.

**TABLE 3.** Contraceptive Availability in Facilities Offering Family Planning and Knowledge and Use Among Married Women of Reproductive Age

**Method**	**Contraceptive Availability in Facilities^a^**		**Contraceptive Knowledge and Use^b^**		
	**% Offer Method**	**% Method Available on Day of Survey**	**% Know of Method**	**% Ever Used Method**	**% Currently Using Method**
Standard Days Method	75	12	64.1	1.4	0.3
Female sterilization	6		77.0	0.7	0.7
Male sterilization	4		56.0	0.2	0.1
Pills	93	71	89.1	15.2	6.4
Intrauterine devices	20	44	54.4	0.8	0.2
Injectables	93	71	91.3	26.1	15.2
Implants	51	49	57.8	2.1	1.6
Male condoms	91	69	98.4	5.9	1.9
Female condoms	35	57	60.2	0.2	0
Emergency contraceptive pills	16	22			

a Source: Rwanda Service Provision Assessment, 2008, Tables A-5.1 and A-5.2.^15^

b Source: Rwanda Interim Demographic and Health Survey, 2008, Tables 5.1, 5.3.1, and 5.4.^16^

During the pilot phase, once women had become aware of SDM, there was sizable demand for it; 23% of new family planning users had chosen SDM during the pilot phase. Although method uptake is expected to be somewhat lower in scale-up sites than in pilot sites, and DHS included sites where SDM had not yet been introduced, the extremely low 0.3% user figure coupled with the seemingly high percentage of facilities offering the method signaled that something was wrong.

The SPA final report revealed that while 75% of facilities reported that SDM was available, CycleBeads were observed in only 12% of facilities—in reality, rendering the method unavailable in most facilities per the program's definition. In the SPA report, SDM method provision was probably defined as having trained providers at the facility and/or having the method listed in the facility service statistics, without considering actual availability of CycleBeads and other package components.

Problems with integrating CycleBeads into the supply chain affected SDM availability and use.

In 2008, the MOH and the Maternal and Child Health Task Force acted on this evidence by tasking the DELIVER Project (a USAID-funded project supporting contraceptive supply systems) to address CycleBeads stockouts at facility levels. The DELIVER Project reviewed the mechanism used by health facilities to order contraceptive supplies (including CycleBeads), instituted a new procedure for requesting urgent supplies, and trained health centers and district pharmacists on contraceptive resupply, particularly for new, underused methods. The scale-up resource team became more vigilant in monitoring stockouts in collaboration with DELIVER Project staff.

About 1 year later, we conducted a facility assessment, in part to determine whether midcourse corrections to the supply chain had resolved the issue with stockouts. The results were encouraging: 90% of facilities offered SDM and only 8% experienced stockouts of CycleBeads in the 3 months preceding the survey (Table 4).

**TABLE 4.** Results From the Rwanda SDM Scale-Up Facility Assessment and Simulated Client Study, April 2009

**Facility Audit (N = 118 facilities)**	**%**
Facilities in which the program manager said that SDM was offered	89.9
Facilities with health providers trained to offer SDM	94.1
Facilities in which CycleBeads were available on day of audit	94.0
Facilities experiencing stockouts of SDM in the 3 months prior to the audit	7.6
**Provider Interviews (N = 155 providers)**	**%**
Trained providers that demonstrated correct knowledge of SDM (on 4 key indicators)	78.0–97.2
Trained providers who offered SDM to at least 1 client in the 3 months preceding the interview	90.8
**Simulated Clients (N = 28 simulated client visits)**	**%**
Received SDM counseling during the visit	78.6
Received CycleBeads during the visit	75.0
Correctly screened for cycle regularity	81.8

Abbreviation: SDM, Standard Days Method.

### Lesson Learned 3. Track and address provider performance to avoid unnecessary medical barriers and ensure fidelity of new method protocols at scale.

A family planning innovation can also lose fidelity during scale up from provider bias and medical barriers. Integration of a new method in a service delivery system requires that providers not only are trained to offer the method but also appreciate its added value, since the providers must adjust their services to include the new method in their program.

During the SDM pilot program, it became clear that many providers doubted whether a fertility-awareness method could be effective. Perhaps in an effort to increase efficacy, some providers applied eligibility criteria that were neither part of the SDM service delivery protocol nor of evidence-based practice, which made the method less accessible. Specifically, some providers required women to monitor their cycle length for several months prior to initiating SDM; to be menstruating at the time they begin using the method; or to have their partner present during the counseling session.

Supervisors corrected such practices during the pilot phase, but this was not feasible during scale up. Early in the scale-up process, MOH district supervision reports provided observational and anecdotal evidence of alterations in the SDM service-delivery protocol. But to document and better define the existence of barriers to SDM adoption in routine service settings, we conducted a special simulated client study in conjunction with the 2009 facility assessment (mentioned under the second lesson learned). The simulated client study was conducted in facilities where providers were not interviewed for the facility assessment.

Simulated clients were women trained to play the role of clients seeking family planning services. They used specially designed client profile scripts that included contraceptive history, partner relationship, and method preference. After each clinic visit, the simulated clients completed a checklist about their experience that included more than 80 objective yes/no indicators regarding what should be included in quality counseling in general, and in counseling on SDM in particular. Items included eligibility screening for using the method, mechanisms of action, use of CycleBeads, correct condom use (for those who wished to use condoms on their fertile days), couple communication about the fertile days, and follow up if there were any problems. This methodology had been validated in a number of previous studies.^17^ To respect principles of informed consent in research, providers in 28 selected facilities from the random sample of facilities participating in the facility assessment consented to be visited by a simulated client sometime over the next year, without knowing the specific date of the visit.

The facility assessment found that 94% of facilities had providers trained to offer SDM, and 94% had CycleBeads in stock. However, the simulated client study showed clearly that providers were creating unnecessary medical barriers to SDM use, thus diminishing method integrity and availability. For example, 21% of simulated clients were not offered SDM despite having the appropriate profile for the method (Table 4). One client who received SDM counseling did not receive CycleBeads at the time of her visit; the provider told her to return when her period started. Others who were given information but not counseling about SDM were also told to return when they got their period or to return with their partner so he could be present for the counseling. Moreover, one provider told a client that she did not offer SDM to her clients because she did not trust the method.

During scale up, providers were imposing unnecessary medical barriers.

The MOH's Maternal and Child Health Task Force and the Family Planning Technical Committee addressed these issues through refresher training and revised supervision protocols in the remaining years of the scale-up process. MOH supervisors worked with providers to become comfortable with offering the new method, including addressing questions of method effectiveness and reducing medical barriers. A small internal study^18^ conducted in 2011 evaluated the effectiveness of the focused supervision approach and found significant improvement. This improvement was confirmed by later supervision visits around the time of the endline evaluation for the scale-up project.[Fig f03]

**Figure f03:**
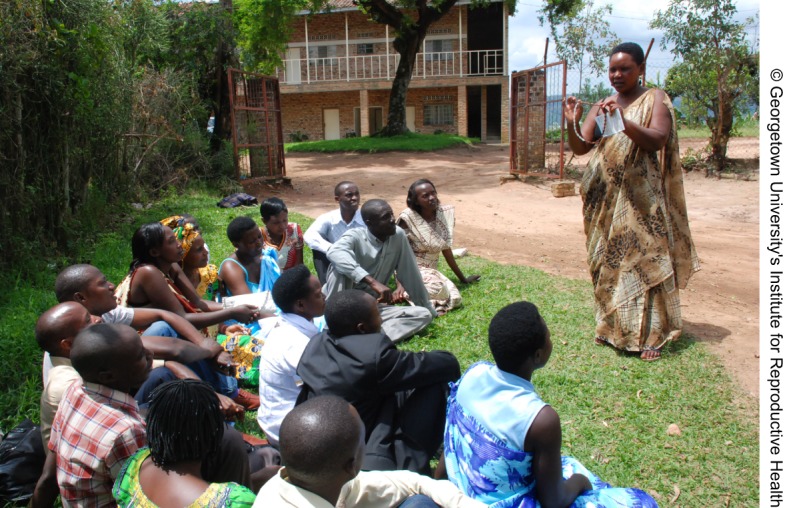
A trainer teaches a group of health care providers how to use CycleBeads, the color-coded string of beads used with the Standard Days Method of family planning.

### Lesson Learned 4: Regularly scan, identify, and address changing environmental influences on scale up.

Since scale-up processes operate within the complex systems in which family planning services are embedded, it is critical to scan environmental factors that may be influencing scale up, such as changes in national leadership or a family planning trend that becomes apparent only through repeated discourse.^19^

Environmental factors, such as change in national leadership, may influence scale up.

A cross-country analysis of factors influencing scale up of SDM in Rwanda and 4 other countries (Democratic Republic of Congo, Guatemala, India [State of Jharkhand], and Mali) revealed the importance of the political and health policy environments; such factors are not typically identifiable via routine monitoring systems because they are often unexpected, imprecise, and come from a host of sources. Therefore, we collected data on environmental factors through other methods including:

- Informal environmental scans to obtain information on social, economic, political, and policy changes but in relatively unstructured ways
- Interviews with staff and scale-up partners to explore their knowledge of the political and policy environments within and external to the national family planning program; this became a regular source of data collection
- Key events timeline, updated semiannually, to track important changes and stakeholder interviews
- FP stakeholder interviews gathered perceptions of forces and factors that might affect scale up from politically connected experts

Assessments from these data sources confirmed that SDM scale up benefited from the Rwandan government's vision of family planning as a crucial national development tool. However, they also revealed the existence of counterforces. In particular, government policy discourse during the scale-up period focused heavily on long-acting and permanent methods which tended to divert attention from SDM. Also, data from environmental scans picked up changes in health financing policies during the second year of scale up. The MOH began promulgating a health-sector performance-based financing (PBF) system about the same time that scale up of SDM was progressing. The system provided incentives for well-performing health centers based on the quantity and quality of specific services they delivered, and while SDM was added to the system in 2009, it was dropped in 2010. Essentially, providers had financial incentives to offer other modern methods but not SDM, thus challenging sustainability of the method.

The government's new focus on long-acting and permanent methods tended to divert attention from SDM.

In response to these environmental obstacles, we positioned SDM among policy makers and influential technical stakeholders as a contraceptive option with unique attributes that filled an important niche in family planning programs. It is a long-acting method since clients can and do continue to use the method for years,^20^ it helps to involve male partners, and it increases women's empowerment through basic understanding of their fertility. In addition, we began one-on-one advocacy efforts with individuals who were influential within the PBF Unit and technical arms of the MOH to provide sound rationales for including SDM in the PBF system. As the 6-year scale-up period ended, this critical issue for sustainability was still unresolved. However, champions had been identified to press the issue further on policy and technical grounds, and it appeared on the way to resolution.

## CONCLUSION

SDM scale up is continuing in Rwanda, as it is in other countries, and the Maternal and Child Health Task Force and other family planning actors are organized to ensure sustainability of method integration.

M&E from multiple sources, including routine monitoring data and impact evaluations as well as special studies and national surveys, played a critical role in scale up by providing timely information for evidence-based decision-making and midcourse corrections to address a number of implementation issues. We learned several important lessons about facilitating nationwide expansion of a new service into an existing FP program and related integration of the service into existing FP support systems. Likewise, as we monitored the process of scale up we learned several important lessons about designing effective M&E systems that recognize complex environments.

First, it is important to apply a systems lens to monitoring and evaluating the scale-up process and for maintaining a focus on sustained availability of quality services over time. We needed data to inform progress in all subsystems relevant to scale up, such as logistics, policies, demand creation, and provider training. This required multiple sources of data as no individual source of data could accurately reveal all the facets of the situation.

Second, data collected for evaluation purposes do play an important role in monitoring for midcourse corrections during scale up. It is important to not conflate impact evaluation with periodic evaluation, which provides timely information throughout a scale-up process. Secondary data sources such as the SPA were very useful in this case, given limitations of funding for primary M&E data collection.

Finally, environmental scanning facilitates the ability of the resource team to address political issues related to scale up in a systematic manner. Timely and accurate information about stakeholder opinions, political events, upcoming policy changes, and resource allocations will increase the effectiveness of resource teams to support the scale-up process.
