# Corrigendum: Igras et al., Systems Approach to Monitoring and Evaluation Guides Scale Up of the Standard Days Method of Family Planning in Rwanda

**DOI:** 10.9745/GHSP-D-17-00359

**Published:** 2017-12-28

**Authors:** Susan Igras, Irit Sinai, Marie Mukabatsinda, Fidele Ngabo, Victoria Jennings, Rebecka Lundgren

**Affiliations:** a Institute for Reproductive Health, Georgetown University, Washington, DC, USA.; b GRM Futures Group, Washington, DC, USA.; c Institute for Reproductive Health, Kigali, Rwanda.; d Ministry of Health [Rwanda], Kigali, Rwanda.

See corrected article.

In the article “Systems approach to monitoring and evaluation guides scale up of the Standard Days Method of family planning in Rwanda” by Susan Igras, Irit Sinai, Marie Mukabatsinda, Fidele Ngabo, Victoria Jennings, and Rebecka Lundgren, which appeared in the May 2014 issue (Volume 2, Number 2) of GHSP, the authors had declared that they had no competing interests. This has been corrected to indicate that Georgetown University, the employer of some of the authors at the time of the writing of the article, owns the patented CycleBeads technology, and that a company owned by a relative of Victoria Jennings has the exclusive license to commercialize the technology. Victoria Jennings has no financial relationship to the company and neither she nor any of the other authors receives royalties or other income related to the licensed technology.

The article has been corrected accordingly.

